# Evidence-based veterinary medicine perception by swine veterinarians: a European survey across diverse practitioner profiles

**DOI:** 10.3389/fvets.2025.1599721

**Published:** 2025-08-29

**Authors:** Charlotte Teixeira Costa, Arnaud Lebret, Clémentine Comer, Nicolas Rose

**Affiliations:** ^1^Rezoolution, Pig Consulting services, Noyal-Pontivy, France; ^2^IRISSO-INRAE, Université Paris-Dauphine, Paris, France; ^3^ANSES, Epidemiology, Health and Welfare Research Unit, Ploufragan, France

**Keywords:** evidence-based, evidence-based veterinary medicine, swine health, Europe, veterinarian

## Abstract

Evidence-Based Veterinary Medicine (EBVM) enhances the quality of care by guiding clinical decisions with robust scientific data, leading to more effective disease management. Evaluating the levels of knowledge and use is crucial for identifying gaps and training needs, ultimately promoting the adoption of evidence-based practices and optimizing herd health and productivity. This study aimed to give an overview of EBVM perception, with the goal of raising awareness of this concept and highlighting reservations they face in applying it to decision-making process in swine veterinary practice. A cross-sectional online survey was conducted among European swine veterinarians. Respondent characteristics and knowledge of EBVM were analyzed using descriptive statistics. Associations between demographic variables (country, specialization) and EBVM knowledge were assessed using Chi-square tests with Yates' continuity correction where appropriate. A significant level of 0.05 was applied. Results showed that 68% of veterinarians were familiar with EBVM, with knowledge levels varying by country (*p* < 0.05) and educational background (p < 0.01). While 82% recognized its practical value, barriers such as limited time, difficulty accessing relevant data, and a lack of decision-support tools hindered implementation. Younger veterinarians and those with less experience expressed more reluctance toward EBVM. Despite these challenges, 90% of respondents believed EBVM improved decision-making, professional confidence, and communication with farmers. However, opinions differed regarding how well EBVM is currently integrated and developed in everyday veterinary practice. These findings highlight the need for enhanced education and structured methodologies to bridge the gap between theoretical knowledge and practical application in herd health management.

## 1 Introduction

Over the past decade, there has been a growing societal expectation that diagnostic and therapeutic decisions made by practitioners are based on evaluations of high-quality scientific literature (1). First described by Sackett et al. (2) as the “conscientious, explicit, and judicious use of current best evidence to make the best possible decision about a patient,” Evidence-Based Veterinary Medicine (EBVM) offers a framework for veterinary practitioners to integrate critical appraisal skills and clinical evidence into their decision-making processes (30). Kochevar and Fajt (30) further elaborated the EBVM process into five key steps: (1) converting the need for information into an answerable question; (2) locating the best evidence to answer the question; (3) critically appraising the evidence for validity, impact, and applicability; (4) integrating the appraisal with clinical expertise and the patient's unique biology, values, and circumstances; and (5) evaluating the effectiveness and efficiency of these steps and seeking ways to improve both the process and clinical outcomes. EBVM is directly inspired by Evidence-Based Medicine (EBM) in human healthcare, though its adoption in veterinary practice is still evolving (3).

While the adoption of EBVM in clinical practice is increasing among veterinary practitioners, it remains an evolving process requiring the development of specific skills and tools (4–6). Incorporating EBVM principles into veterinary education is essential to equip practitioners with the necessary competencies for evidence-based decision-making. For example, Huntley et al. (7) conducted an international survey, including veterinarians primarily working in small animal practice across the UK, USA, Canada, and Australia. They reported that EBVM awareness often originated during veterinary school or university, underlining the importance of education in promoting EBVM principles. For instance, young veterinarians and those who have recently completed further training tend to be more familiar with EBVM principles. (7). However, regardless of age, some veterinarians may feel less confident engaging with scientific research methods due to limited training in epidemiology, statistics, or evidence appraisal during their veterinary education (8).

While previous research has addressed EBVM awareness and practices among veterinarians in general or in companion animal medicine, very few studies have explored its application in the context of herd health management. This represents a significant gap, given the increasing emphasis on evidence-based practices in animal production systems. Compared to individual animal medicine, herd health management introduces unique challenges for EBVM implementation. Swine veterinarians often operate within complex, multifactorial production environments that involve collective decision-making, time constraints, economic pressures, and the need to balance population-level outcomes with individual animal welfare. These factors complicate the straightforward application of research findings and require a broader and more integrated approach to evidence use.

Furthermore, few studies have explored veterinarians' knowledge of EBVM practices, revealing varying levels of understanding. While most practitioners are aware of the concept, many struggle with its practical application in daily practice (9, 10). EBVM was originally introduced in individual animal veterinary medicine, where evidence-based practices follow a structured, unidirectional flow of information from research to assessment and ultimately to clinical application (11). This model, rooted in randomized controlled trials (RCTs), prioritizes methodological rigor and clearly defined clinical endpoints. However, as Karriker (11) also noticed, this approach becomes more complex in the context of production animal medicine, especially swine health. Blinded RCTs—considered the gold standard of clinical research—are particularly difficult to implement in pig barns due to ethical, logistical, and economic constraints. As a result, the type of evidence generated in swine medicine is often perceived as weaker, even if it remains relevant and valuable for field application. This reality influences not only the availability of high-quality evidence but also veterinarians' confidence in its applicability to herd-level decision-making. Unlike individual animal medicine, which focuses on single-patient diagnoses and treatments, herd health management relies on a population-level approach that incorporates multifactorial influences and collective decision-making. This shift in decision-making necessitates adapting EBVM principles to population-level dynamics, economic constraints, and the involvement of multiple stakeholders (12). The application of these principles varies across production systems, such as pig farming. Furthermore, there is limited consensus on core outcome measures in swine medicine, which hinders the comparability of studies and the development of a robust evidence base. As highlighted by Sargeant et al. (13), establishing standardized core outcome sets would improve consistency across trials and strengthen the evidence supporting EBVM in swine practice. Moreover, the demanding nature of managing health across numerous pig farms leaves veterinarians with limited time to engage with scientific literature, attend continuing education courses, or participate in conferences. When assessing how veterinarians worldwide are accessing information, a recent study showed that both practitioners and non-practitioners consult a wide array of journals and electronic resources (14). Examples of such electronic resources include online databases, professional forums, and veterinary-specific websites. This suggests that veterinarians are actively consulting a variety of journals and digital resources to stay informed. If this information is made accessible and practical, veterinarians could effectively translate and communicate evidence-based health management strategies directly to producers. However, beyond access to information, the practical implementation of EBVM principles requires a structured approach. This includes the ability to critically appraise scientific evidence, integrate it with clinical expertise and farm-specific constraints, and apply it through standardized decision-making frameworks. Encouraging veterinarians to adopt a methodological approach, such as formulating answerable clinical questions, systematically searching for evidence, and objectively evaluating study outcomes, could help close the gap between theoretical knowledge and practical application in herd health management.

In this context, the objectives of this study were to: (1) assess the level of knowledge of European swine veterinarians regarding EBVM and how it impacts their practice, (2) raise their awareness of this concept, and (3) evaluate the obstacles European swine veterinarians face in applying EBVM to swine health management strategies in practice.

## 2 Material and methods

### 2.1 Survey design

The survey was designed to evaluate veterinarians' knowledge of EBVM and their level of engagement in its application (see Supplementary File 1). It consisted of 67 questions, integrated into a broader questionnaire on porcine reproductive and respiratory syndrome virus (PRRSV) and related practices, totaling 197 questions. The questionnaire consisted predominantly of multiple-choice questions (~85%), complemented by short-answer questions (~15%) that allowed respondents to provide more detailed feedback or explanations where appropriate. The questionnaire was piloted with three swine veterinarians from different European countries to ensure clarity and comprehension. Based on their feedback, minor changes were made to the wording and structure of some questions, particularly multiple-choice items. The estimated time to complete the entire questionnaire was ~30 min. Most questions were closed-ended to enhance the comparability of responses. A brief introduction provided the context for the study. Emphasis was placed on the anonymity of responses and the importance of this survey in improving and facilitating swine veterinary practice. The first question aimed to determine the level of knowledge of veterinarians regarding EBVM. Subsequent questions explored key aspects of EBVM, including barriers to its adoption, perceived usefulness, commonly used tools, and existing knowledge gaps. Additionally, an open-ended question allowed respondents to share their concerns or fears regarding EBVM implementation. Veterinarians were asked to rank both the perceived relevance of EBVM and their perception of how developed or established EBVM practices are within their daily clinical work. Furthermore, nine additional questions were included to identify the tools and data sources veterinarians rely on in their professional activities. Lastly, a section of the questionnaire was dedicated to collecting general demographic and professional information about the respondents, such as gender, age, degree, type of workplace (size, proportion of veterinarians and technicians), country, and years of experience.

### 2.2 Data collection and analysis

The target population included vet practitioners in Europe who dedicated at least 50% of their practice to pig health. To ensure respondent eligibility, an open-ended question at the beginning of the survey asked: “What percentage of your working time is dedicated to swine health?” Only responses indicating 50% or more were included in the analysis. All respondents in the final dataset met this requirement. Additionally, the survey was distributed exclusively through professional networks involved in swine health, helping to ensure the relevance of the sample. Open-ended responses collected in the survey were analyzed using thematic content analysis. Two researchers independently coded the qualitative data to identify recurring themes related to barriers, concerns, and perceptions of EBVM among swine veterinarians. Discrepancies in coding were resolved through discussion to reach consensus. This approach ensured a rigorous and repeatable analysis of the qualitative feedback provided by respondents. The questionnaire was developed as an online survey using Sphinx iQ3 (Version 8.2.2). It was distributed via email. All contact emails were collected through the French Association for Pig Health (ANSP), the French Interprofessional Pork Council (INAPORC), the European College of Porcine Health Management (ECPHM), colleagues, and, in some cases, through contacts within pharmaceutical companies. Data collection took place between October 2nd, 2023 and March 1st, 2024, after extending the initial deadline by 1 month. Respondents' characteristics and EBVM knowledge were analyzed quantitatively. The responses' dataset was transferred to a Microsoft Excel V.14.0.6 (2010, Microsoft) spreadsheet for data management. Descriptive statistics, including frequencies and percentages, were calculated to summarize respondents' characteristics and their knowledge of EBVM. Associations between categorical variables such as country, age, gender, and EBVM knowledge were assessed using Chi-square tests with Yates' continuity correction where appropriate. A significance level of 0.05 was applied. For theses analysis, no missing data was encountered in the questions analyzed, as most questions were closed-ended with forced responses or based on predefined lists, ensuring complete datasets for statistical analysis.

## 3 Results

### 3.1 Study sample

The survey was sent to 657 veterinarians in 24 countries across Europe. A total of 108 veterinarians from 22 countries filled in the questionnaire, which corresponds to a response rate of ~16.6%. The characteristics of respondents are summarized in Table 1. Most respondents were male (60%), salaried employees (54%), with over 10 years of professional experience (80%). Approximately 30% of respondents were from France, with others representing various European countries. Over half of the respondents reported having either a national or European specialization (53%). Although the target was veterinary practitioners who spent at least 50% of their time monitoring pig farms, our sample was composed almost completely of swine veterinarians dedicating 100% of their time to pig practice.

**Table 1:** Main characteristics of survey respondents (*n* = 108 European pig veterinarians).

**Variable**	**Category**	**Sample (*n* = 108) Number (Percentage)**
Gender	Male	63 (58.3%)
	Female	45 (41.7%)
Age	< 30 y/o	8 (7.4%)
	30–39 y/o	24 (22.2%)
	40–49 y/o	37 (34.3%)
	50–59 y/o	27 (25.0%)
	≥60 y/o	12 (11.1%)
Status	Salaried practitioner	58 (53.7%)
	Independent practitioner	50 (46.3%)
Number of employees in the company	Less than 10	34 (31.5%)
	Between 10 and 50	42 (38.9%)
	More than 50	32 (29.6%)
Proportion of veterinarians among the total number of employees (in %)	≤ 20%	16 (14.8%)
	21–79%	36 (33.3%)
	≥80%	56 (51.9%)
Experience of veterinarians in pig health	Less than 10 years	23 (21.3%)
	More than 10 years	85 (78.7%)
Other diploma	European specialist	11 (10.2%)
	National specialization	47 (43.5%)
	Other^1^	5 (4.6%)
	None	45 (41.7%)
Country	Austria	5 (1.9%)
	Belgium	4 (4.0%)
	Croatia	3 (3.1%)
	Denmark	10 (5.2%)
	England + Scotland	1 (1.7%)
	Finland	1 (1.0%)
	France	34 (29.3%)
	Germany	2 (2.3%)
	Greece + Cyprus	4 (3.7%)
	Hungary	5 (6.4%)
	Italy	4 (4.1%)
	Luxembourg	1 (0.7%)
	Malta	1 (1.1%)
	The Netherlands	9 (7.6%)
	Norway	1 (1.2%)
	Poland	2 (2.6%)
	Portugal	2 (1.9%)
	Republic of Ireland	4 (6.2%)
	Romania	1 (1.6%)
	Spain	6 (6.2%)
	Sweden	5 (6.4%)
	Switzerland	3 (3.7%)

^1^The category “other” includes other diploma with the same level as osteopathy, agricultural diploma, etc.

### 3.2 Basic knowledge about EBVM

**Figure 1:**
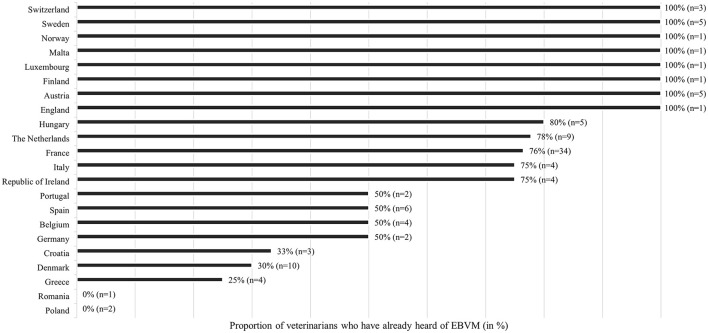
EBVM knowledge level by country of practice (*n* = 108).

Sixty-eight percent of veterinarians answered “yes,” meaning they had already heard of the EBVM concept before being contacted (Figure 1). Despite the limited number of veterinarians in some countries, we observed that 100% of veterinarians who completed the questionnaire in Poland and Romania were unfamiliar with EBVM. Similarly, a large proportion of veterinarians in Greece (75%, *n* = 3), Denmark (70%, *n* = 7), and Croatia (67%, *n* = 2) had never encountered the concept. In other countries, at least 50% of respondents were already aware of EBVM. Our results showed a significant association between veterinarians who knew the concept and the country of the respondent (*p* < 0.05) or the fact of having a European or national specialist diploma (*p* < 0.01). While, most veterinarians became aware of EBVM through veterinary schools (42%, *n* = 31), others learned about the concept through training programs and conferences (16%, *n* = 12), professional associations (7%, *n* = 10), scientific literature (19%, *n* = 14), or colleagues (8%, *n* = 6).

### 3.3 European veterinarians' opinions on the utility of EBVM

More than 82% of veterinarians recognized the practical value of the EBVM approach, even if they were unfamiliar with the concept itself. However, 19 out of 108 veterinarians considered EBVM to be either not useful or only conditionally useful, depending on factors such as the type of pathology, the specific challenges encountered in the field, or the availability of reliable scientific data. Many respondents highlighted the variable applicability of EBVM, noting that while it can be a valuable complement in uncommon cases, its relevance is more limited in routine practice. One practitioner remarked, “*Sometimes we apply things without scientific proof that they work, we simply don't have enough data.”* Others (*n* = 19) emphasized the importance of pragmatic considerations, with one stating, “*If it benefits the farmer, that's what matters.”* Additionally, a certain mistrust toward the European scientific community was expressed, with some veterinarians questioning the reliability of the research available. Some practitioners also admitted using EBVM selectively, as a way to validate their existing practices rather than as a framework for change. Despite some reservations expressed by a minority of respondents, the majority acknowledged that the usefulness of EBVM depends on the clinical context, with its relevance varying from moderate in routine cases to more critical in complex or uncommon situations. This contextual nuance emerged particularly from the open-ended responses explaining why some veterinarians viewed EBVM as conditionally useful or less applicable in daily practice.

### 3.4 Definitions and features regarding EBVM according to swine veterinarians

**Figure 2:**
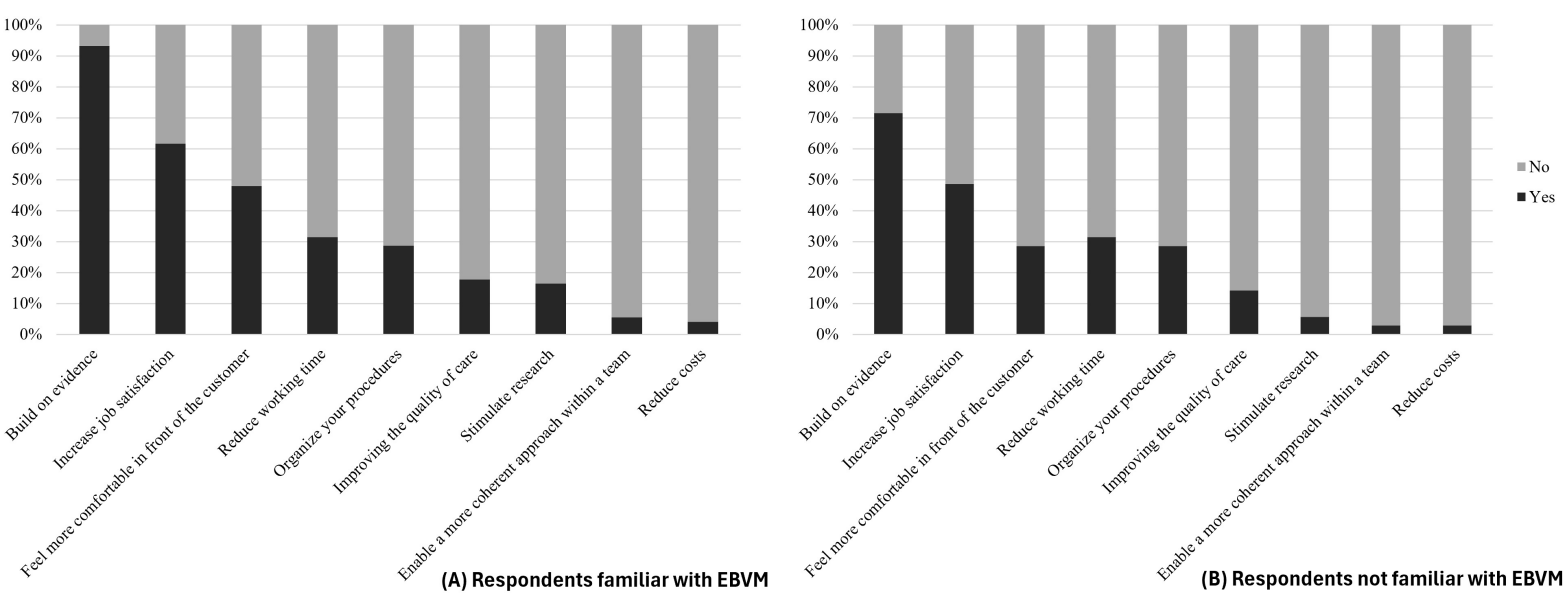
Opinions of swine veterinarians on the definition of EBVM depending on whether they had previously heard of the concept **(A)** or not **(B)** (*n* = 108). **(A)** shows responses from veterinarians familiar with EBVM, while **(B)** presents responses from those unfamiliar with the concept.

For the majority of respondents, the EBVM approach was primarily seen as a way to rely on evidence to make better decisions, with nearly 90% agreeing on this point, regardless of their level of knowledge (Figure 2). Prior familiarity with EBVM significantly influenced respondents' perceptions, with those already acquainted with the concept more likely to recognize its practical value and benefits in clinical practice compared to those unfamiliar with it (*p* < 0.01). This suggests that experience or education on EBVM enhances appreciation of its usefulness. Additionally, more than 50% of respondents viewed EBVM as a tool to increase job satisfaction, while over 40% believed it helped them feel more confident when interacting with farmers. This latter perception was strongly influenced by employment status (p < 0.01): 86% of independent practitioners felt that EBVM made them more comfortable in their role, compared to only 14% of salaried veterinarians.

### 3.5 Obstacles and barriers regarding EBVM use

#### 3.5.1 Swine veterinarians' requirements

**Figure 3:**
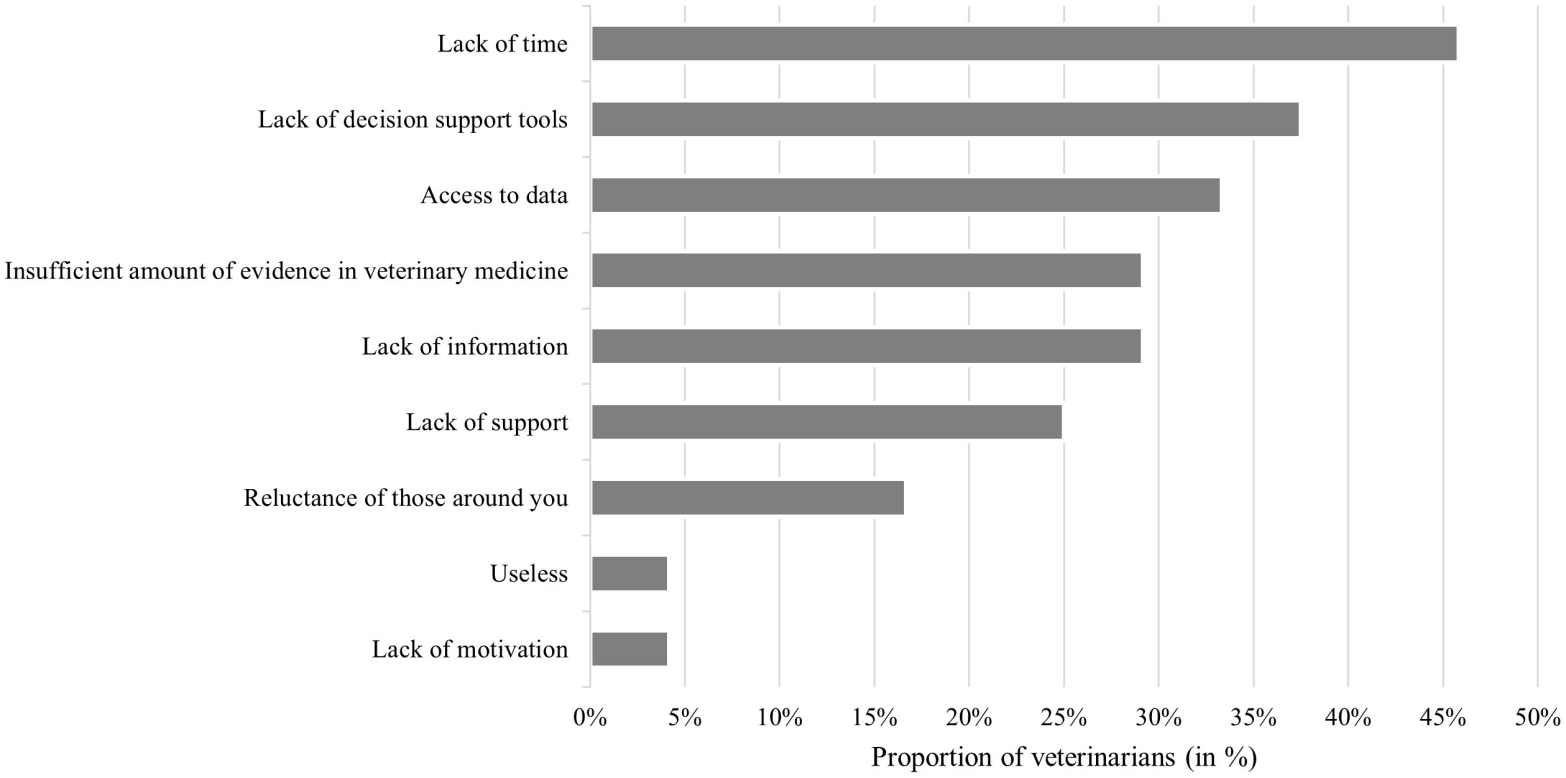
Barriers to EBVM implementation in daily practice (*n* = 108).

A total of 24 out of 108 veterinarians (22%) reported encountering obstacles when trying to implement EBVM in their practice. Among them, over 46% believed that EBVM requires more time than they can afford (Figure 3). Additionally, around 33% assumed that accessing relevant data is difficult, with many reporting challenges in retrieving scientific articles. Moreover, nearly 40% of respondents felt that there is a lack of decision-support tools to effectively apply EBVM. Finally, approximately 25% found EBVM challenging to implement due to a lack of support or resistance from colleagues, making its use uncomfortable in their daily practice.

#### 3.5.2 Impact of age and veterinary experience on concerns about EBVM

Resistance to EBVM was strongly associated with a veterinarian's limited experience in swine health, namely among those with < 10 years of practice (*p* < 0.001). Indeed, 48% (*n* = 11) of veterinarians with < 10 years of experience expressed reluctance toward using EBVM, compared to only 15% of those with more than 10 years of experience. In the same way, age also played a significant role: younger veterinarians (< 30 years old) were more hesitant to use EBVM in practice, with 60% expressing concerns, compared to < 30% in older age groups (*p* < 0.001).

### 3.6 Impact of EBVM on practitioners' confidence

**Figure 4:**
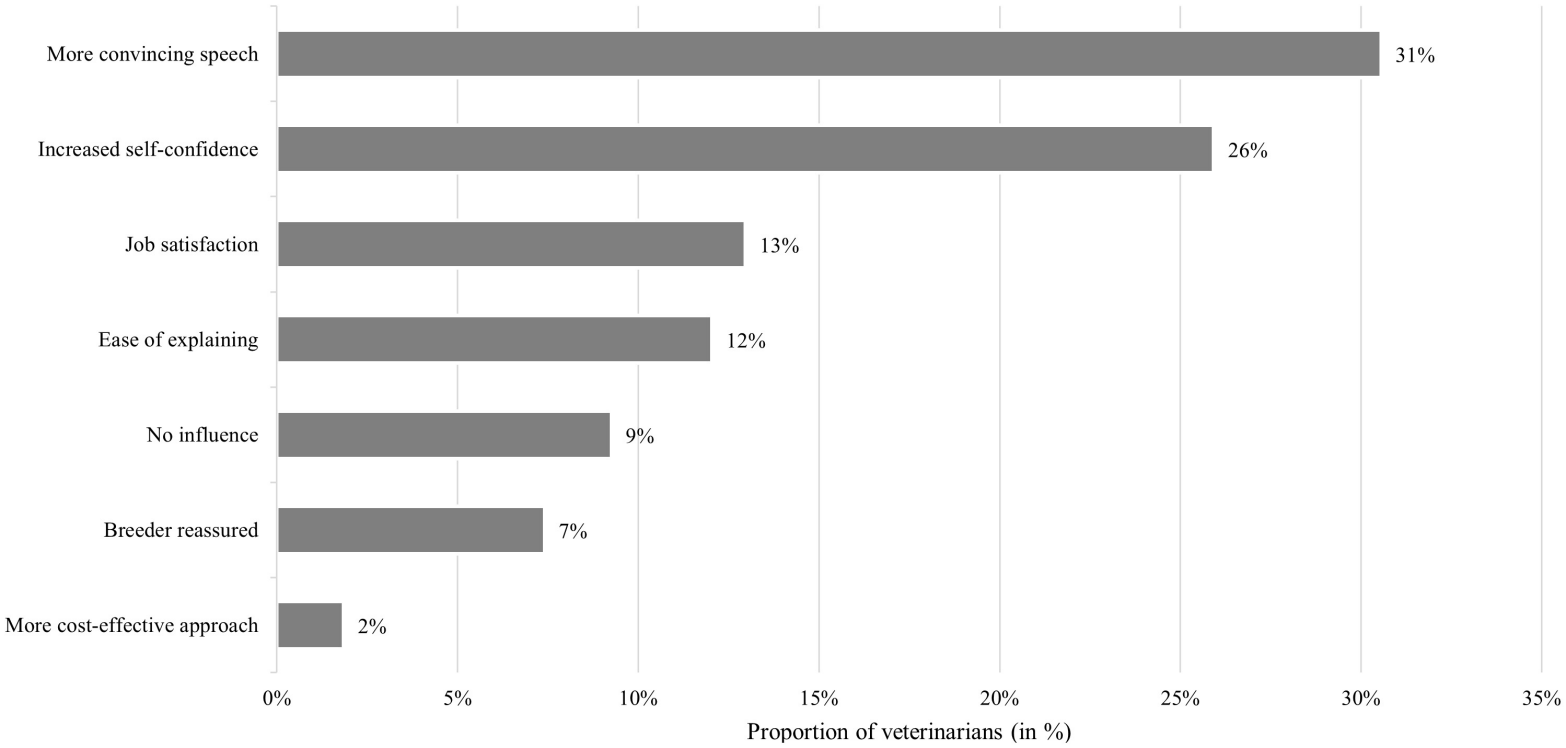
Veterinarians' opinions regarding the influence of EBVM on farmer's relationship (*n* = 108).

Over 90% of all respondents felt that their relationship with farmers was easier to establish when they could present evidence-based support for their recommendations. Among these respondents, 73% (*n* = 72) reported monitoring regularly the farms under their care. Additionally, over 30% felt that their arguments became more persuasive as a result (Figure 4). This trend appeared to be influenced by gender, with a higher proportion of men reporting greater confidence in their communication compared to women (*p* < 0.10). Similarly, veterinarians working in larger companies tended to express higher self-confidence compared to those working in smaller companies (*p* < 0.10).

### 3.7 Is EBVM sufficiently developed in swine practice?

**Figure 5:**
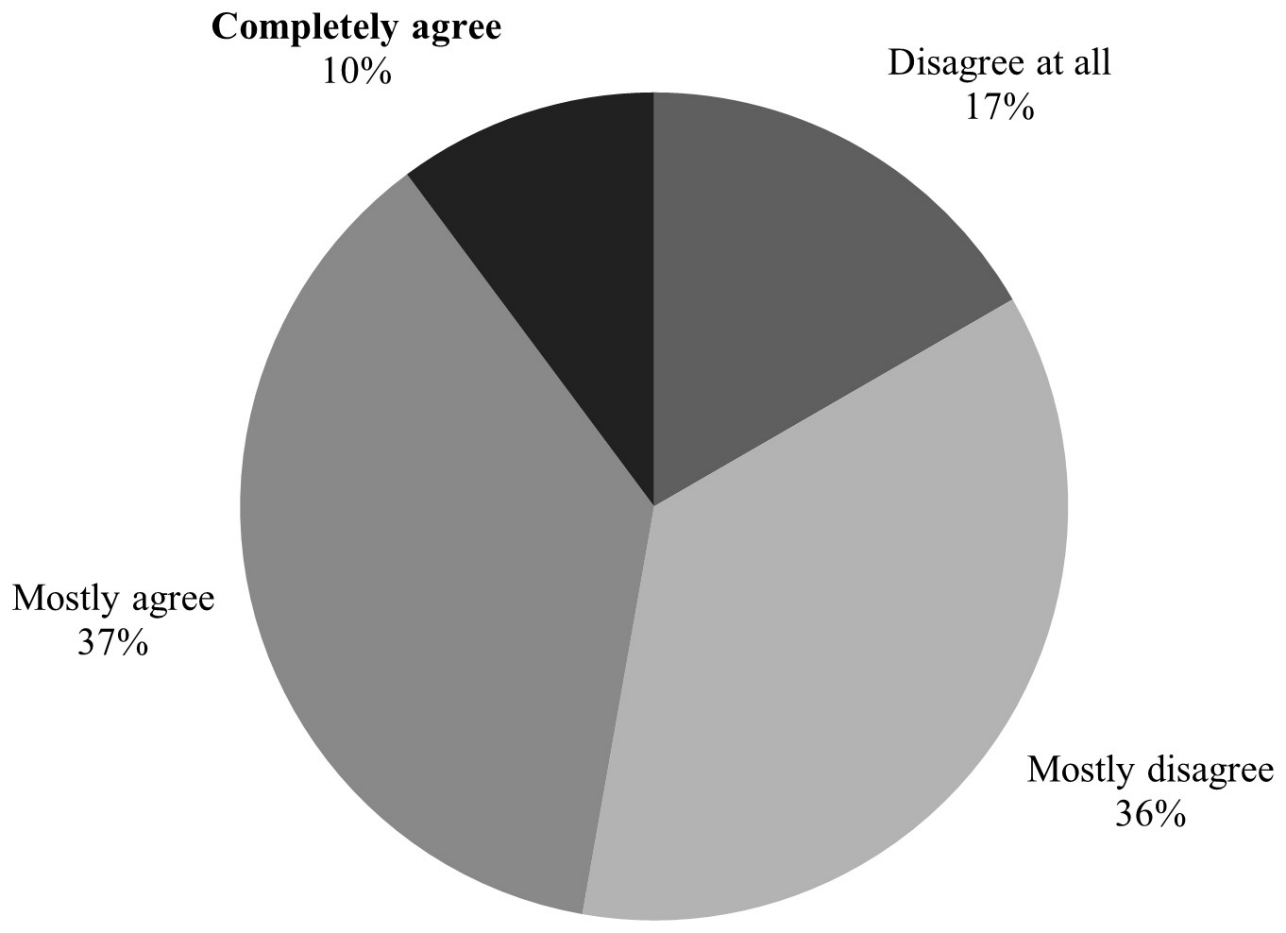
Swine practitioners' opinion on the development of EBVM in their practice (*n* = 108)

Responses regarding the development of the EBVM concept in practice were mitigated (Figure 5). More than 47% (*n* = 51) of respondents believed that the approach is sufficiently developed, while 53% disagreed. Among the 108 veterinarians surveyed, 18 (17%) felt that EBVM is not developed at all in practice, whereas 11 (10%) completely agreed that it is sufficiently established. Additionally, women tended to be more likely to agree that EBVM is adequately developed in practice (p < 0.10).

## 4 Discussion

This study provides new baseline data on European swine veterinarians' knowledge and perceptions of EBVM. To our knowledge, it is the first European-wide study focusing on veterinarians who dedicate at least 50% of their practice to pig health. While EBVM has been widely discussed in veterinary literature (15–17, 29), few studies have specifically explored its application in rural or swine-focused veterinary contexts. Compared to these studies, our findings reveal a lower level of EBVM awareness among swine practitioners, despite a generally positive attitude toward its principles. This discrepancy could be due to limited integration of EBVM into swine-focused curricula or continuing education programs. In contrast to studies where EBVM was well established among clinicians (18, 19), our results suggest that swine veterinarians face unique structural and informational barriers, reinforcing the need for targeted educational and decision-support interventions in this field.

This survey represents the first phase of a broader project on the EBVM concept and its application to veterinary decision-making for PRRSV control. This context may have influenced participants' responses by framing EBVM primarily within the scope of infectious disease management. As such, respondents may have viewed EBVM more through the lens of disease control strategies, potentially underrepresenting its perceived usefulness in other aspects of swine veterinary practice. It was designed as a European-wide study targeting veterinarians with at least 50% of their practice dedicated to pig health. Email contact was chosen as the primary communication method to maximize reach. Although response rates in other studies using similar outreach methods were generally higher (10, 14), our survey remains satisfactory given its European scope and the specificity of its target audience—a relatively small population of swine practitioners. However, the results must be interpreted with caution. Moreover, although the study was conducted across 24 countries, the number of respondents per country varied considerably, with some countries having only one or two participants. As a result, our findings should not be interpreted as representative at the national level. The sampling strategy was based on voluntary participation through professional networks, which may have led to selection bias particularly favoring veterinarians with a pre-existing interest in EBVM or PRRSV. This self-selection may influence the perceived level of EBVM awareness or motivation for its use.

While our sample was diverse in terms of gender, status, age, and experience, nearly 30% of respondents were based in France, suggesting a potential overrepresentation of this group. Furthermore, due to the absence of comprehensive demographic data on the European swine veterinarian population, we cannot confidently assess the overall representativeness of our sample. Nevertheless, we found no published data to assess it. Additionally, there is a potential selection bias, as respondents may have been those with a greater interest in this issue, such as practitioners actively dealing with PRRSV and seeking decision-making tools for disease control. Despite these biases, the survey provides valuable insights into pig veterinarians' knowledge of EBVM. Our results reveal substantial knowledge gaps regarding EBVM among swine veterinarians across Europe. The relatively low familiarity with the concept may reflect disparities in veterinary curricula or continuing education opportunities. Interestingly, the countries with the highest awareness may reflect stronger academic emphasis on EBVM or more active involvement in professional training networks. These findings support the idea that EBVM dissemination remains uneven across Europe and highlight the need for harmonized educational efforts at the European level. Notably, education was the primary source of knowledge on EBVM, reinforcing previous findings that formal education plays a crucial role in informing veterinary practitioners (8, 20, 21). For instance, Block (2024) recently reported that EBVM remains insufficiently emphasized in veterinary curricula. Although the study did not focus specifically on European institutions, the findings likely reflect a broader trend that may also apply to veterinary education in Europe, thereby contributing to the ongoing challenges in integrating EBVM into daily practice. Similarly, Keay et al. (22) conducted an international survey of swine veterinarians identifying barriers and knowledge gaps related to epidemiology, statistical methods, and research interpretation, which likely impact evidence-based veterinary practice across various countries, including Europe. Moreover, our survey revealed that respondents with additional diplomas (e.g., in agriculture or osteopathy) tended to be better informed about EBVM. This outcome might be expected, as these individuals may have broader educational backgrounds and greater exposure to scientific literature and professional networks (13, 23). Our results highlighted the crucial role of conferences, seminars, and professional training events in fostering EBVM awareness. This is particularly evident among specialists, as the European College of Porcine Health management (ECPHM) guidelines mandate that graduates demonstrate their professional competence by practicing evidence-based veterinary medicine (The European College of Porcine Health management, Constitution and Bylaws, article 4, section 10, ECPHM 2024). Thus, our survey results highlight the importance of continuously educating swine practitioners on evidence-based decision-making to enhance their clinical practices.

Our findings indicate an association between reservations toward EBVM and demographic factors such as age and years of professional experience, which are closely related, making it difficult to separate their individual effects. While it is often assumed that more experienced veterinarians may be less receptive to new approaches, such as EBVM, our data also suggest that younger professionals encounter challenges in applying EBVM. Specifically, younger and less experienced veterinarians more frequently reported lack of time as a significant barrier to implementing EBVM in their daily work. These challenges may be influenced by workplace structure: early-career veterinarians often handle high-volume, routine cases and have limited decision-making authority. These structural and organizational factors could reduce their opportunities to engage with evidence-based approaches, despite having recently acquired theoretical knowledge. This complexity points to the need for further investigation into how demographic, organizational, and educational factors interact in shaping EBVM adoption in veterinary practice.

Our study also highlighted the need for swine veterinarians to better understand and properly implement the EBVM approach in their daily practice. Our findings suggest that developing decision-making tools and facilitating access to relevant data could enhance their ability to integrate this concept into their work. Improving veterinary practices, particularly in terms of facilitating diagnosis and clinical decision-making, will depend on both the specific disease and the availability of relevant literature on the topic. Therefore, gathering additional input from veterinarians regarding their specific needs and gaps is essential to support better decision-making when faced with a disease (24).

Moreover, veterinarians are recognized as key actors in transmitting health knowledge to farmers (25–27). Given their generally positive perception of EBVM, they could serve as an effective knowledge transfer channel between researchers and farmers. However, our results indicate that practitioners often lack time to stay up to date with recent literature, highlighting the need for researchers to explore more efficient ways of formalizing and disseminating information. Future improvements should focus on strengthening communication and knowledge transfer between research and veterinary practice. Since EBVM remains relatively unknown, ongoing research projects aim to better understand the obstacles to its implementation and its practical benefits. Regular updates on these findings should be shared with practitioners to ensure they remain informed about the method and its advantages. To this end, an initial factsheet summarizing European swine veterinarians' perspectives on EBVM will be developed and distributed.

Additionally, our survey was part of a broader project aimed at evaluating the role of the “Evidence-Based Medicine” concept in veterinary decision-making within organized production systems, specifically in the context of controlling PRRS in pig farming. Thus, our present results should be analyzed within the context of veterinarians' attitudes toward the EBVM concept and its application to PRRSV control. Indeed, the questionnaire investigated more deeply three other main areas: (i) Regulatory framework (the country-specific regulations governing PRRSV control and the resources available for its management). (ii) Disease management knowledge, including understanding the interactions within PRRSV control systems. (iii) Decision-making tools [among other thing, technology-assisted decision-making tools as described by Zhou et al. (28)] including digital platforms, data analytics, and monitoring technologies that support PRRSV prevention and control efforts. In this context, veterinarians' responses may likely be influenced not only by their level of knowledge of EBVM but also by their individual PRRSV approach. Further studies will be necessary to determine whether veterinarians are both willing and able to implement PRRSV control and surveillance plans using tools derived from the EBVM approach.

## 5 Conclusion

Our baseline study revealed significant knowledge gaps regarding EBVM among swine practitioners that need to be addressed. Targeted educational initiatives should be implemented to raise veterinarians' awareness of EBVM and its practical applications. Before launching a PRRSV control plan based on EBVM-driven decision-making tools, further research is required to assess the specific needs for this objective. Such data would provide valuable insights into veterinarians' perspectives, enabling a more customized approach and adapted specifically to different practitioner profiles (for example, early-career vs. experienced veterinarians). Supporting early-career veterinarians with mentoring, practical resources, and simplified access to evidence summaries could improve their ability to implement EBVM in daily practice. Finally, collaboration between veterinary faculty, professional associations, and industry stakeholders is essential to ensure that training initiatives are aligned with field realities and help create optimal conditions for evidence-based disease control on pig farms. This, in turn, would help guide efforts to overcome specific barriers and create optimal conditions for effective disease control on pig farms.

## Data availability

The raw data supporting the conclusions of this article will be made available by the authors, without undue reservation.
