# Supplementary material for: Evidence-based veterinary medicine perception by swine veterinarians: a European survey across diverse practitioner profiles
Source: Front Vet Sci. 2025 Aug 29;12:1599721. doi: 10.3389/fvets.2025.1599721 (PMC12426164; doi:10.3389/fvets.2025.1599721)
Supplement: Supplementary file 1 [file Data_Sheet_1.pdf]

Supplementary Material

Supplementary Data

Survey designed to evaluate veterinarians' knowledge of EBVM and their level of engagement in its application.

1_General data

1. Gender

Female ; Male ; Other

The response is mandatory.

2. Age

The response is mandatory. The response must be more than 18.

3. In what year did you obtain your veterinary degree?

Please right the years in format yyyy

The response is mandatory. The response must be more than 1950.

4-5. What diploma(s) did you obtain in addition?

National Specialization ; ECPHM diploma ; Other

A question "If Other, please specify" is associated with this question.

5. If other, specify :

The response is mandatory.

6. Practice as:

Liberal practitioner ; Employee

The response is mandatory.

7-8. If employee, specify:

Industry ; Industries pharmaceutiques ; Health association ; Independent firm ; Civil servant ; Administration ; Other

The response is mandatory. A question "If Other, please specify" is associated with this question.

The question is relevant only if type_poste among "Employee".

8. If other, specify :

The response is mandatory.

9. What is the size of the company in which you operate?

Less than 5 employees ; Between 5 and 10 employees ; Between 10 and 50 employees ; More than 50 employees

The response is mandatory.

10-11. Which professions are found in your company?

Technicians ; Veterinarians ; Other

The response is mandatory. A question "If Other, please specify" is associated with this question.

You can check multiple boxes.

11. If other, specify :

The response is mandatory.

12. What is the proportion of veterinarians within your structure?

The response is mandatory. The response must be included between 0 and 100.

The question is relevant only if prop_tech_veto among "Technicians;Other".

13. How many years of swine practice experience do you have?

Less than 5 years ; Between 5 and 10 years ; More than 10 years The response is mandatory.

14. Are you working at:

Full time ; Part time

The response is mandatory.

15. What is your employment rate?

The response is mandatory. The response must be included between 0 and 100.

The question is relevant only if contrat among "Part time".

16. Among your activities, what proportion represents the share dedicated to pig farming?

The response is mandatory. The response must be included between 0 and 100.

In which sector of intervention (geographical area) do you mainly operate?

The response is mandatory.

[17-17]. Main sector of intervention:

Majority area

The response is mandatory.

[18-18]. In which country is it located?

Majority area

Germany ; England ; Austria ; Belgium ; Bulgaria ; Cyprus ; Croatia ; Denmark ; Spain ; Estonia ; Finland ; France ; Greece ; Hungary ; Ireland ; Italy ; Latvia ; Lithuania ; Luxemburg ; Malta ; Norway ; The Netherlands ; Poland ; Portugal ; Czech Republic ; Romania ; Slovakia ; Slovenia ; Sweden ; Swiss

2_EBVM and veterinary practices

19-20. Do you usually work as a team within your structure?

Yes ; No ; It depends

The response is mandatory. A question "If Other, please specify" is associated with this question.

21. if 'It depends' specify:

The response is mandatory.

22-23. At what stage(s) do you think teamwork is necessary?

Modalities for implementing an diagnosis ; Analysis of technical and economic data ; Help in monitoring farms ; Interpretation of laboratory analysis results ; Other

Classify your chooice according to the importance for you

The response is mandatory. A question "If Other, please specify" is associated with this question.

Order a maximum of 5 responses.

The question is relevant only if equipe_travail among "Yes;It depends".

24. If other, specify:

The response is mandatory.

25. Within your company, do you hold meetings to develop common procedures (Guidelines)?

Never ; Rarely ; Occasionally ; Quite often ; Very often

The response is mandatory.

Weight:1 ; 2 ; 3 ; 4 ; 5

26-27. Which topic(s) are these meetings about?

Zootechnical issues (feed, genetics, etc.) ; Sanitary issues ; Herd Case Report ; Development of new tools ; Bibliography sharing ; Other

The response is mandatory. A question "If Other, please specify" is associated with this question.

You can check multiple boxes.

The question is relevant only if equipe_travail among "Yes;It depends".

28. If other, specify:

The response is mandatory.

29. Do you interact with other peers (outside your company)?

Yes ; No

The response is mandatory.

30-31. Rank in order of importance these interactions?

Pharmaceutical laboratory veterinarians ; Pharmaceutical laboratory technicians ; Veterinarians from

diagnostic laboratory ; Technicians from diagnostic laboratory ; Other veterinarians ; Other technicians ; Administration ; Other

Order only the three main interlocutors

The response is mandatory. A question "If Other, please specify" is associated with this question.

Order a maximum of 3 responses.

The question is relevant only if pairs among "Yes".

31. if other, specify:

[32-36]. How often do you interact with these peers?

32. Veterinarians and pharmaceutical laboratory technicians

33. Veterinarians and technicians from diagnostic laboratory

34. Other veterinarians

35. Other technicians

36. Administration

Rarely ; Occasionally ; Quite often ; Very often

The response is mandatory.

Weight:1 ; 2 ; 3 ; 4

[37-39]. How often :

37. Do you read new publications?

38. Do you attend congresses/seminars?

39. Do you participate in training?

Express your answer as a percentage of your working time

The response is mandatory.

40-41. How are you informed of the latest studies published on swine pathologies?

Colleagues ; Networks (LinkedIn, Research Gate, ...) ; Active bibliographic search with alerts ; Subscription to a bibliographic search service ; Training ; Other

The response is mandatory. A question "If Other, please specify" is associated with this question.

You can check multiple boxes.

41. If other, specify :

The response is mandatory.

42-43.Rank in order of importance the type of evidence that you think most impacts your decisions?

Case report ; Meta-analysis ; Randomized clinical trial ; Cohort study ; Expert advice ; Editorials ; Books ;

Other

The response is mandatory. A question "If Other, please specify" is associated with this question.

Order a maximum of 8 responses.

43. If other, specify :

44. Situate the degree of importance you give to peer review communications:

Not peer-reviewed -- Peer-reviewed

The response is mandatory.

[45-47]. For each line, indicate the modality that have the greatest impact on your decision-making:

Personal experience, A = Scientific articles, G = Company guidelines, C = Congresses, CE = External advis

45. Diagnostic procedure

46. Disease control protocol

47. Disease eradication procedure

EP ; A ; G ; C ; CE

The response is mandatory.

Tick a maximum of 4 boxes.

3_Knowledge and perception of the EBVM

48. Before we contacted you, had you ever heard of the concept of 'Evidence-Based Medicine' (EBM)?

Yes ; No

The response is mandatory.

49. How did you get aware about EBM?

The question is relevant only if connaissance_ebm among "Yes".

50-51. In your opinion, what is an EBM approach?

Based on evidence ; Enable a more cohesive approach within a team ; Increase job satisfaction ; Feel more comfortable with the customer ; Lower the costs ; Organize your procedures (diagnosis, treatment, analyses, etc.) ; Improving the quality of care ; Reduce working time ; Stimulate research ; Other

The response is mandatory. A question "If Other, please specify" is associated with this question.

You can check multiple boxes.

51. If other, specify:

52-53. Do you think this approach is useful for helping decision-making in your practice?

Yes ; No ; It depends

The response is mandatory. A question "If Other, please specify" is associated with this question.

53. If 'It depends' specify:

The response is mandatory.

54. Do you have any reluctances or obstacles in using this approach?

Yes ; No

The response is mandatory.

55-56. Why don't you use this method?

Lack of time ; Lack of motivation ; Useless, waste of time ; Lack of information ; Difficult access to data (many sites, costs, etc.) ; Insufficient amount of evidence in veterinary medicine ; Lack of decision support tools ; Reluctance of the entourage (colleagues for example) ; Lack of support ; Other

The response is mandatory. A question "If Other, please specify" is associated with this question.

You can check multiple boxes.

The question is relevant only if freins_ebm among "Yes".

56. If other, specify:

The response is mandatory.

57-58. In all the farms that you monitor, do you regularly monitor technical and economic performance?

Yes ; No ; It depends

The response is mandatory. A question "If Other, please specify" is associated with this question.

58. If 'It depends' specify:

59-60. What types of data do you analyze?

Analysis of economic data ; Analysis of technical and reproductive data ; Monitoring of slaughter classification indicators (condemnations, classification of carcasses...) ; Analysis of weight, ADG, consumption... ; Other

The response is mandatory. A question "If Other, please specify" is associated with this question.

You can check multiple boxes.

The question is relevant only if perf_tech among "Yes;It depends".

60. If other, specify :

The response is mandatory.

61. Do you have tools to facilitate data analysis?

By 'tools' we mean computer tools for example

Yes ; No

The response is mandatory.

62-63. Which?

Excel ; R ; Python ; SAS ; Professional calculators ; Other

The response is mandatory. A question "If Other, please specify" is associated with this question.

You can check multiple boxes.

The question is relevant only if outils among "Yes".

63. If other, specify:

64. In your opinion, does having evidence to bring change your relationship with the farmer?

Yes ; No

The response is mandatory.

65-66. In what sense?

Increase in self-confidence ; Professional satisfaction ; More convincing speech ; Reassured farmer ; Easy of explaining ; Stress reduction ; More cost effective approach ; Other

Rank your first four answers in order of importance

The response is mandatory. A question "If Other, please specify" is associated with this question.

Order a maximum of 4 responses.

The question is relevant only if relation_eleveur among "Yes".

66. If other, specify:

The response is mandatory.

67. In your opinion, is EBVM a sufficiently developed approach in daily practice?

Totally disagree ; Tend to disagree ; Tend to agree ; Totally agree

The response is mandatory.

Weight:1 ; 2 ; 3 ; 4

Would you like the results of this study to be communicated to you?

Yes ; No

The response is mandatory.

Please enter the email address to which you would like the results sent to you:

The response is mandatory.

The question is relevant only if com among "Yes".
